# Application of the suture needle retrograde threading method in scleral fixation of intraocular lenses

**DOI:** 10.1186/s12886-023-03258-9

**Published:** 2023-12-19

**Authors:** Zhou Zhou, Gang Yao, Guangyi Huang, Haibin Zhong, Qi Chen, Ke Yang, Shan Zhong, Min Li, Fan Xu

**Affiliations:** Institute of Ophthalmic Diseases, Guangxi Academy of Medical Sciences & Department of Ophthalmology, the People’s Hospital of Guangxi Zhuang Autonomous Region & Guangxi Key Laboratory of Eye Health & Guangxi Health Commission Key Laboratory of Ophthalmology and Related Systemic Diseases Artificial Intelligence Screening Technology, Nanning, 530021 Guangxi People’s Republic of China

**Keywords:** Scleral fixation, Intraocular lenses, Surgical techniques, Ophthalmology

## Abstract

**Background:**

Here we described a new threading technique for the universal fixation of any posterior chamber intraocular lens (IOL).

**Methods:**

Twenty-seven eyes of 27 patients whose surgery done by Surgeon A with the needle-guided method or the suture needle retrograde threading (SNRT) method for intrascleral IOL fixation were enrolled in the first group. Thirty-four eyes of 34 patients whose surgery done by Surgeon A, Surgeon B or Surgeon C with the SNRT method for intrascleral IOL fixation were grouped into three sub-groups by surgeon. Information regarding age, sex, best-available visual acuity (BCVA), intraocular pressure (IOP), past ophthalmological history, threading time (from puncturing to externalizing suture) and complications during and after the surgery were gathered.

**Results:**

The analysis showed that the threading time was less in the SNRT group than needle-guided group by Surgeon A. There was one eye with suture needle slipping from the guide needle when guiding out of the eye. The threading procedure was completed one time without suture ruptures or loop slippage in the SNRT group operated by Surgeon A. And using the SNRT method, Surgeon A, Surgeon B, and Surgeon C did not show any significant difference in threading time. No complications (e.g., vitreous hemorrhage, hyphemia, retinal detachment, suprachoroidal hemorrhage, or hypotony) were observed during surgery or postoperatively in all cases. No leakage occurred at the site of the puncture after the operation.

**Conclusions:**

The described technique appears to be a safe, simple, easy-to-learn, and universal surgical method, which is suitable for various types of IOLs.

**Supplementary Information:**

The online version contains supplementary material available at 10.1186/s12886-023-03258-9.

## Background

Various surgical techniques have been utilized for the placement of intraocular lens (IOL) in eyes without support from the posterior capsule. These techniques include implanting IOL in the anterior chamber, iris-fixated IOL, and scleral-fixated IOL implantation via suture-based or suture-free intrascleral fixation [1]. Rarely performed, anterior chamber IOL implantation is associated with high rates of complications, including glaucoma and corneal decompensation. Meanwhile, the effectiveness of iris claw lens implantation depends on the presence of sufficient iris support and a pupil of normal size and shape, limiting its applicability in cases of traumatic sphincter injury [2]. The use of GORE-TEX suture fixation can lead to potential complications, including suture exposure and subsequent secondary endophthalmitis [3]. Therefore, Scleral fixation of the IOL is still the main approach used by both cataract and vitreoretinal surgeons.

In recent years, there have been two dominant patterns in the advancement of scleral fixation methods. The Yamane flanged haptic scleral fixation technique, which has gained widespread acceptance, is a sutureless procedure used to secure the haptics of the three-piece IOL within the sclera. The mechanical stability of it could be long-lasting, as the flanges created by heat prevent the conjunctiva from opening and exposing the sclera [4]. The Veronese technique, utilizing the FIL SSF Carlevale IOL with flexible transscleral plugs, has recently employed a comparable fixation principle [5]. Nevertheless, there are constraints on the selection of intraocular lenses using both methods. The Yamane method only allows for the use of three-piece IOLs, whereas the Veronese method is specifically designed for the authors’ IOL model, which is not widely available for purchase.

The use of sutures for IOL fixation makes their selection more feasible. The approaches for suture- guiding including ab interno and ab externo (needle-guided and built-in sutures, most commonly). However, some challenges arise during certain steps of this surgery. Complications, such as retinal detachment and hemorrhage, may arise due to the ab interno technique’s use of a blind maneuver and the unpredictable positioning of haptics. Insufficient placement of the polypropylene needle into the lumen of the 27-gauge needle occurs in the needle-guided method [6]. In this scenario, when the 27-gauge needle is removed from the eye, the polypropylene needle may detach from the 27-gauge needle and drop into the vitreous cavity. Introducing the suture into the lumen of the 27-gauge needle can be challenging in the inherent suture technique, and there is a risk of the needle bevel severing it upon entry.

Therefore, we introduce a new threading method that creates less disturbance to the vitreous body, has low operation requirements, has a short learning curve, has no direction requirement for the incision, and can be used for various forms of IOL suture fixation.

## Materials and methods

### Participants

Enrolled in this study were patients who had the IOL surgery with intrascleral fixation performed from January 2021 to November 2022. The first group included 41 eyes of 41 patients whose surgery done by Surgeon A with the needle-guided method or the SNRT method for intrascleral fixation of IOL. The second group included 60 eyes of 60 patients whose surgery done by Surgeon A, Surgeon B or Surgeon C with the SNRT method for intrascleral fixation of IOL. The protocols were carried out in accordance with the Declaration of Helsinki and received approval from the Hospital ethics committee. All patients provided written consent.

### Data collection and definitions

Information regarding age, sex, best-available visual acuity (BCVA), intraocular pressure (IOP), past ophthalmological history, threading time (from puncturing to externalizing suture) and complications during and after the surgery were gathered. The observational metrics encompassed the threading time, pre- and post-surgical BCVA, IOP, and complications. Before the operation and during the final follow-up appointment, the BCVA was assessed using the Snellen chart. IOP was measured by non-contact tonometry (Topcon, Japan). In other studies, hypotony was characterized as an IOP below 5 mmHg, while hypertension was described as an IOP above 25 mmHg during any follow-up visit after surgery, using definitions similar to those used previously [7]. For statistical analysis, the BCVA were converted to logMAR equivalents by taking the logarithm of the minimum angle of resolution (logMAR). Like other studies, a visual acuity of counting fingers and hand motions was converted to a logMAR of 1.98 and 2.28, respectively [8].

### Statistical analysis

In this study, the learning curve was quantitatively evaluated using the cumulative summation (CUSUM) analysis, which was derived from a previous technique [9, 10]. The formula utilized in this analysis is as follows:

$${\text{CUSUM}}=\sum_{i=1}^{n}\left(xi-u\right)$$

$xi$ represents the actual operation time for each case, while $u$ represents the average operation time for a specific group of patients. The cases were arranged in chronological order, and the learning curve was constructed by cumulatively summing the disparity between the actual operation time and the average operation time for each case. The inclination of the CUSUM curve signifies the trajectory of the learning outcome, and the juncture at which the inclination transitions from positive to negative is regarded as the threshold at which the learning curve is surpassed [9, 11].

The IBM® SPSS® Statistics software (version 27.0 for Windows; SPSS Inc., Chicago, IL, USA) was utilized for conducting statistical analysis. The normal distribution of variables was confirmed through the examination of skewness, kurtosis, and the Kolmogorov–Smirnov test. Variables were compared using either parametric or non-parametric tests, depending on the distribution of the data. A significance level was determined with a P value less than 0.05.

### Surgical technique of the SNRT method

**Fig. 1:**
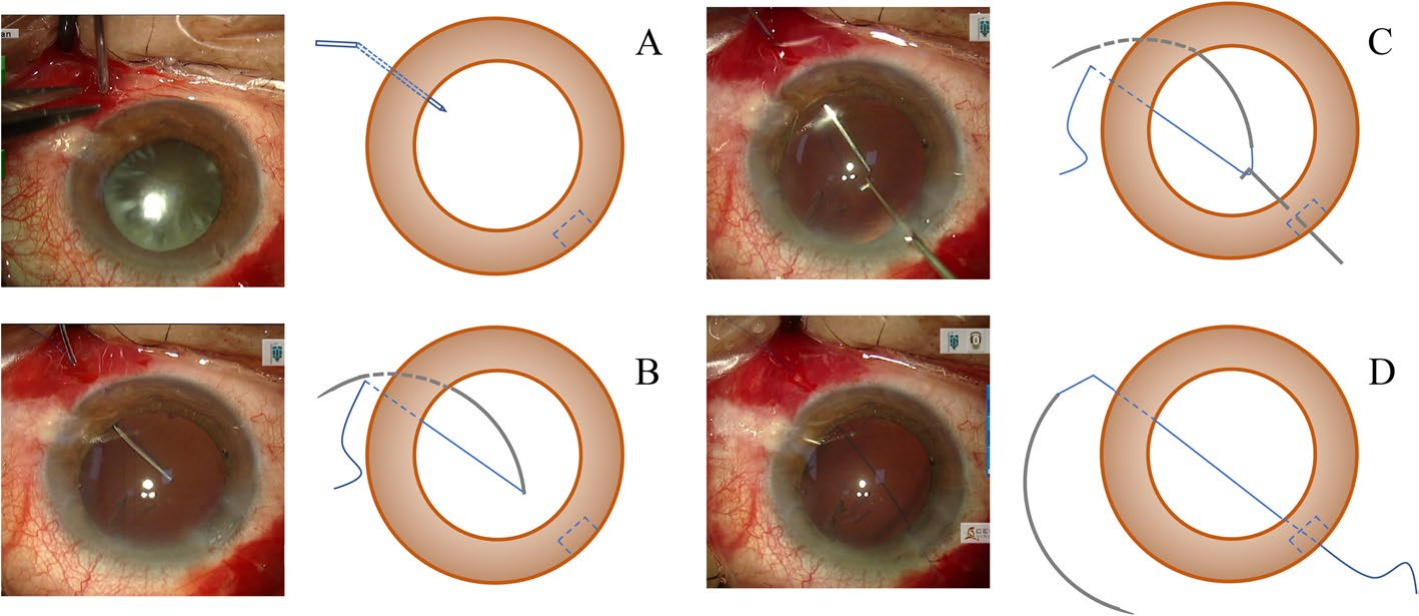
Key steps of the SNRT method. **A** A 23-gauge needle was employed to puncture the posterior chamber. **B** The retrograde insertion of the 9–0 polypropylene suture's tip into the eye began at the puncture site. **C**, **D** An IOL adjustment hook was utilized to extract the suture from the corneal incision. SNRT stands for suture needle retrograde threading; IOL stands for intraocular lens

We utilized retrobulbar anesthesia by injecting 2 ml of 2% lidocaine from Shanghai Zhaohui Pharmaceutical Co., Ltd. As previously planned, puncture marks were created 2 mm behind the limbus. Using a 23-gauge needle, the posterior chamber was punctured at the location of the designated point (Fig. 1A). Using the 9–0 polypropylene suture needle tip (2452L, MANI, Japan), it was inserted into the eye by reversing it through the puncture point (Fig. 1B). The suture was externalized from the corneal incision using an IOL adjustment hook (Fig. 1C, D). The above steps allowed for safe suture placement on both sides of the corneal puncture. After that, the lens was injected into the eye while the remaining haptic was left outside and the haptic of the lens were secured with a knot. Finally, the other haptic of the IOL was dialed into the eye and the suture was tightened and adjusted to the IOL optical center. These aforementioned surgical procedures can be observed in the accompanying surgical video (Supplementary video). Subsequently, the procedure of transscleral suture fixation was carried out in accordance with the previously provided instructions. The needle-guide method was carried out as described [6].

## Results

### Patient baseline characteristics

This study included 61 patients who had intra-scleral IOL fixation in 61 eyes. They were divided into two groups based on different intraoperative guidance methods. The experimental group used the SNRT method proposed in this study, while the control group used the needle-guided method. Table 1 summarizes the characteristics of both groups.

**Table 1:** Baseline characteristics

Variable	Control group	The SNRT group			*P* value
	Surgeon A (*N* = 12eyes)	Surgeon A (*N* = 15eyes)	Surgeon B (*N* = 18eyes)	Surgeon C (*N* = 16eyes)	
Age (years)	51.92 ± 3.22	56.33 ± 4.29	55.72 ± 3.62	49.88 ± 5.46	0.6702
Sex (male/female)	(5 / 7)	(8 / 7)	(9 / 9)	(7 / 9)	NA
Preoperative BCVA (logMAR)	1.05 ± 0.12	1.06 ± 0.10	0.98 ± 0.07	1.12 ± 0.09	0.5458
Preoperative IOP (mmHg)	16.38 ± 0.68	14.34 ± 0.60	14.46 ± 0.62	15.81 ± 0.84	0.1290
Past ophthalmological history					
AMD	1	0	1	2	NA
Glaucoma	0	1	0	0	NA
Retinal detachment repair by PPV	1	2	0	0	NA
Diabetic retinopathy	0	0	1	0	NA
Silicone Oil implantation status	1	1	2	2	NA

*NA* stands for not available, *BCVA* refers to best-available visual acuity, *IOP* stands for intraocular pressure, *AMD* stands for age-related macular degeneration, and *PPV* stands for pars plana vitrectomy

### Efficacy and safety evaluation of the SNRT method

**Fig. 2:**
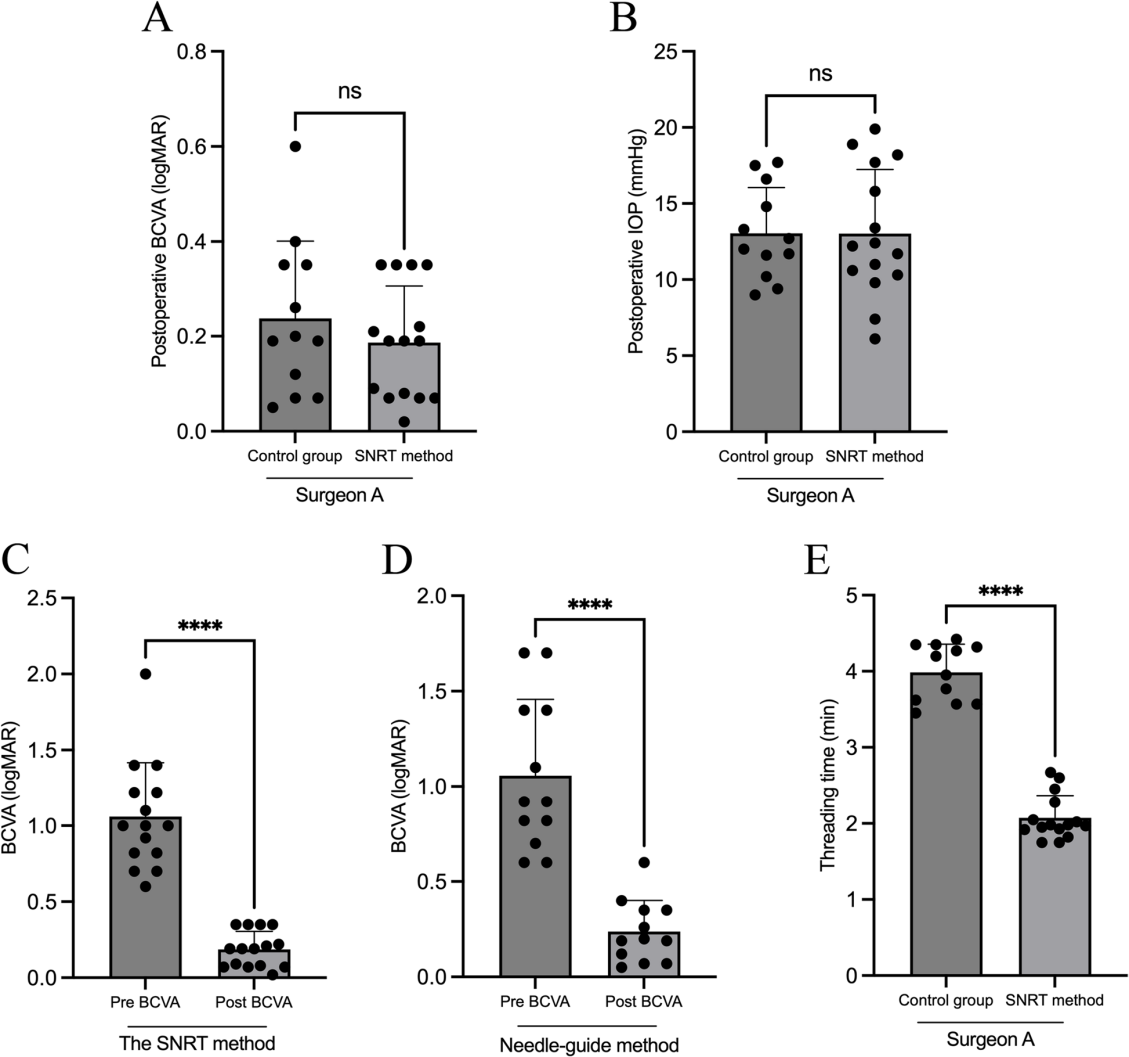
The SNRT method demonstrates favorable surgical outcomes and safety measures, thereby enhancing surgical efficiency. **A**, **B** Comparison of postoperative BCVA and IOP between surgeon A’s surgery using the needle-guided method and the SNRT method. **C**, **D** Comparison of preoperative and postoperative BCVA between two groups of patients using the needle-guided method and the SNRT method. **E** Comparison of threading time between surgeon A’s surgery using the needle-guided method and the SNRT method. SNRT stands for suture needle retrograde threading; BCVA stands for best-available visual acuity; IOP stands for intraocular pressure; ns stands for not significant; **** stands for *P* < 0.0001

A comparative analysis was performed on two groups of patients operated by surgeon A to assess the surgical outcomes of different leads. The evaluation included the needle-guided method and the SNRT method, focusing on BCVA, IOP, and complications. The findings showed no statistically significant difference in postoperative BCVA and IOP between the two patient groups, as illustrated in Fig. 2A and B. Nevertheless, a notable enhancement in both groups was noticed when comparing the BCVA before and after the surgery (Fig. 2C, D).This implied that our proposed SNRT method can yield comparable postoperative outcomes to the needle-guided method.

To further evaluate the safety of the SNRT method, we monitored postoperative ocular complications in both groups of patients for 3 months. Among these, vitreous hemorrhage, hyphemia, retinal detachment, suprachoroidal hemorrhage, hypotony and incisional leakage were the potential complications we focused on monitoring. Follow-up showed that neither group of patients developed any of these complications within 3 months of surgery. This was in line with previous observations. Therefore, the above results indicated that the SNRT method has a good safety profile for intra-scleral IOL fixation surgery.

### The SNRT method improves surgical efficiency

Increasing surgical efficiency plays an important role in enhancing patient outcomes. To investigate the impact of the SNRT method on the surgical efficiency of the intra-scleral IOL fixation procedure, a comparison was made between the threading time (from puncturing to externalizing suture) required by surgeon A when employing the needle-guided method and the SNRT method. And the findings revealed that surgeon A required a significantly lower average threading time when employing the SNRT method compared to needle-guided method (Fig. 2E). The implementation of the SNRT method resulted in a 47.96% reduction in average threading time, thereby substantially enhancing the efficiency of the scleral IOL fixation procedure.

### Learning curve analysis of the SNRT method

**Fig. 3:**
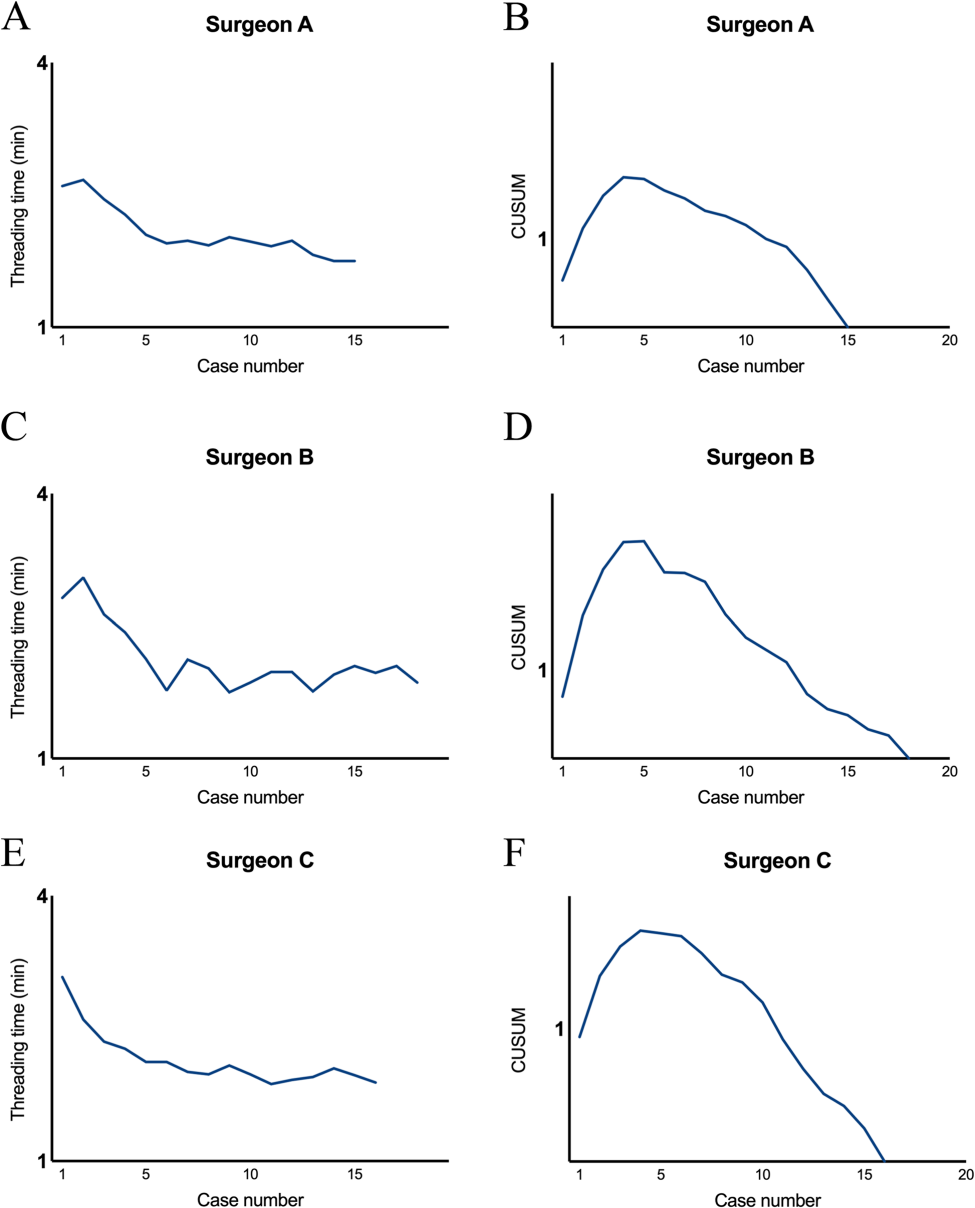
Learning curve analysis of the SNRT method. **A**, **B** Threading time curves and CUSUM curves for surgeon A using the SNRT method. **C**, **D** Threading time curves and CUSUM curves for surgeon B using the SNRT method. **E**, **F** Threading time curves and CUSUM curves for surgeon C using the SNRT method. SNRT stands for suture needle retrograde threading; CUSUM stands for cumulative summation

To further assess the learning curve of the SNRT method, we collected data on the threading time of surgeries performed with this method by surgeons with different levels of experience. Surgeons A, B and C, with more than 15, 10 and 5 years of ophthalmic surgical experience respectively, were included in the study. The results showed that the average threading time for surgeons A, B, and C using the SNRT method was 2.08 ± 0.08, 2.12 ± 0.09, and 2.14 ± 0.08 min, respectively, with no statistical difference (*P* = 0.8526). Furthermore, the slopes of the corresponding learning curves showed that they peaked at the 5th, 6th and 5th cases, respectively, indicating that the learning curves for surgeons A, B and C to perform surgeries using the SNRT method were 4, 5 and 4 cases, respectively (Fig. 3A-F). Based on these results, we concluded that the SNRT method has a short learning curve and can be mastered by young ophthalmologists in a relatively short cycle.

## Discussion

The absence of adequate capsular support for IOL placement poses a distinctive surgical challenge. Scleral IOL fi xation is primarily used for cases involving subluxation or dislocation of the lens or IOL–capsular bag complex and aphakia with inadequate capsular support due to any cause. Research findings indicate that the adoption of sutureless fixation techniques for IOL is progressively gaining popularity. For example, the successful utilization of a novel CM T-flex IOL, which effectively addresses the limitations associated with conventional three-piece IOL, particularly in aniridia eyes with a filtering bleb, underscores the significance of further exploration and consideration of sutureless IOL fixation approaches in future studies [12, 13]. However, transscleral suture fixation possesses a broader scope of practical applications owing to its enhanced tolerance of intraoperative materials. Consequently, our study concentrates on the technical enhancement of said fixation method.

The approaches to scleral fixation include ab interno and ab externo, and the key point of it was suture guidance. Performing the ab interno method is more instinctive compared to the ab externo method and is simpler to execute when combined with penetrating keratoplasty surgeries. Nevertheless, this action is a blind maneuver linked to retinal detachment, hemorrhage, and unpredictable placement of the lens haptics.

Currently, the ab externo approach is a mainstream surgical method. Lewis introduced the idea of ‘docking’ in 1991, which involves inserting a straight needle on a 10–0 polypropylene suture into a 28-gauge needle at a 180° angle [14]. This technique ensures a consistent final position of the IOL in the ciliary sulcus, while also preventing injury to the ciliary process or retina, unlike the ab interno method. However, it requires good coordination with both hands and has an extensive learning curve. There is also the possibility of an intraoperative suture slip leading to intraocular injury [7]. In addition, during the operation, the eyeball may be compressed due to the long span or an odd angle between the two docking needles, resulting in complications, such as vitreous loss, suprachoroidal hemorrhage, and hypotony.

To solve the above problems, some scholars have proposed using a built-in suture in the syringe needle (similar to a sewing machine) that goes through the sclera into the eye [8]. However, due to ophthalmic viscosurgical device (OVD) or liquid adhesion during the operation and the suture itself being too thin, it is inconvenient to send the suture into the syringe needle. In addition, the needle may damage or amputate sutures. Therefore, an alternative is the use of special forceps (e.g., retinal forceps [15], a ciliary sulcus guide [16], or a suture thread inserter [17]) to place sutures in the eye through the scleral puncture. However, special devices indicate some restrictions to its widespread.

To solve the existing problems, a simple suture needle retrograde traversing method was proposed in this study. We used a 23-gauge needle to puncture the sclera first and then placed the end of a 9–0 polypropylene suture needle backward into the eye through the puncture point. After that, the suture was externalized from the corneal incision using an IOL adjustment hook (Fig. 1).

The benefits of the SNRT method are multiple. First, it is a simple and quick method for IOL scleral fixation. Compared with other techniques, our method eliminates the difficulty of traversing sutures into the eye. It does not require a guide needle or a suture built into the syringe needle. In addition, due to its simple operation, this technique can be applied to 360° of the limbus, and there is no special requirement for the position of the corneal incision, which is conducive to different directions of IOL fixation.

Second, it is safe. The needle does not need to pass through the anterior or posterior chamber. By only passing through the sclera, the likelihood of iris injury and vitreous hemorrhage caused by damage to the ciliary process is minimized. Meanwhile, it can minimize the risk of multiple anterior segment manipulations. By using a closed system for fixation, we maintained IOP control and avoided complications from severe fluctuation of IOP, such as retinal detachment, choroidal detachment, and suprachoroidal hemorrhage.

Third, this method is a dependable surgical procedure that is appropriate for novices and surgeons who have limited experience. Our technique is minimally invasive and minimizes awkward manipulations. Performing each step of this method is uncomplicated and only necessitates the use of one hand, making it easy to perform with a short learning curve.

Fourth, our approach is universal for different patients’ situations and different types and sizes of needles and threads. In addition, the suture can not only be pulled completely out of the eye to perform the knot at the end of the suture but can also be pulled part of the suture out to form the extraocular loop. This technique is suitable for single- or double-thread sutures and can be matched with different knotting methods, such as cow knots and clove knots, which widens the scope of possible applications. Finally, this technique requires no special instruments and is more economical and suitable for popularization and application.

Notably, there were some limitations to the current study. The suture has a certain elasticity, and the tip of the IOL adjusting hook is cylindrical with a T-shape. Therefore, it may not be easy to externalize the suture using the IOL adjustment hook. In this situation, we can use a blunt chop instead. Additionally, pulling too fast may damage the suture so we need to pay attention to the resistance when drawing the suture, and draw out it slowly at a constant speed. Finally, when shorter stitches are used, an effective inner eye loop may not be formed, resulting in difficulty in threading. Therefore, the surgeon must select a syringe needle that matches the size of the suture needle in order to insert the needle end smoothly. The primary objective of this study was to evaluate the suture guidance technique, SNRT, in the intra-scleral IOL fixation. It is important to note that different fixation methods could potentially affect the overall surgical outcome. Therefore, future research should aim to evaluate the surgical outcome of the SNRT in combination with different fixation methods.

## Conclusions

In conclusion, we believe that the SNRT method is a safe, simple, easy-to-learn, and universal surgical method that is suitable for all kinds of IOL fixation techniques. Further studies with more cases and various instances are needed to support the initial results and to compare them with other threading techniques to assess their applicability.

### Supplementary Information

**Additional file 1: Supplementary video.** A surgical video demonstration of the SNRT method. The procedure consists of the initial creation of punctures on both sides of the cornea, the subsequent retrograde guidance according to the SNRT method and finally the implantation and fixation of the IOL.

## Data Availability

The datasets used and/or analysed during the current study available from the corresponding author on reasonable request.

## Abbreviations

IOL: Intraocular lens
SNRT: Suture needle retrograde threading
BCVA: Best-available visual acuity
IOP: Intraocular pressure
logMAR: Logarithm of the minimum angle of resolution
CUSUM: Cumulative summation
AMD: Age-related macular degeneration
PPV: Pars plana vitrectomy
OVD: Ophthalmic viscosurgical device
